# Immunosenescence and Inflamm-Aging As Two Sides of the Same Coin: Friends or Foes?

**DOI:** 10.3389/fimmu.2017.01960

**Published:** 2018-01-10

**Authors:** Tamas Fulop, Anis Larbi, Gilles Dupuis, Aurélie Le Page, Eric H. Frost, Alan A. Cohen, Jacek M. Witkowski, Claudio Franceschi

**Affiliations:** ^1^Research Center on Aging, Graduate Program in Immunology, Faculty of Medicine and Health Sciences, University of Sherbrooke, Sherbrooke, QC, Canada; ^2^Singapore Immunology Network (SIgN), Biopolis, Agency for Science Technology and Research (A*STAR), Singapore, Singapore; ^3^Department of Biochemistry, Graduate Program in Immunology, Faculty of Medicine and Health Sciences, University of Sherbrooke, Sherbrooke, QC, Canada; ^4^Department of Infectious Diseases and Microbiology, Faculty of Medicine and Health Sciences, University of Sherbrooke, Sherbrooke, QC, Canada; ^5^Department of Family Medicine, Faculty of Medicine and Health Sciences, University of Sherbrooke, Sherbrooke, QC, Canada; ^6^Department of Pathophysiology, Medical University of Gdańsk, Gdańsk, Poland; ^7^Italian National Research Center on Aging, Department of Experimental Pathology, University of Bologna, Bologna, Italy

**Keywords:** inflamm-aging, immunosenescence, immunometabolism, immune-adaptation, immunoremodeling, longevity, healthspan

## Abstract

The immune system is the most important protective physiological system of the organism. It has many connections with other systems and is, in fact, often considered as part of the larger neuro–endocrine–immune axis. Most experimental data on immune changes with aging show a decline in many immune parameters when compared to young healthy subjects. The bulk of these changes is termed immunosenescence. Immunosenescence has been considered for some time as detrimental because it often leads to subclinical accumulation of pro-inflammatory factors and inflamm-aging. Together, immunosenescence and inflamm-aging are suggested to stand at the origin of most of the diseases of the elderly, such as infections, cancer, autoimmune disorders, and chronic inflammatory diseases. However, an increasing number of immune-gerontologists have challenged this negative interpretation of immunosenescence with respect to its significance in aging-related alterations of the immune system. If one considers these changes from an evolutionary perspective, they can be viewed preferably as adaptive or remodeling rather than solely detrimental. Whereas it is conceivable that global immune changes may lead to various diseases, it is also obvious that these changes may be needed for extended survival/longevity. Recent cumulative data suggest that, without the existence of the immunosenescence/inflamm-aging duo (representing two sides of the same phenomenon), human longevity would be greatly shortened. This review summarizes recent data on the dynamic reassessment of immune changes with aging. Accordingly, attempts to intervene on the aging immune system by targeting its rejuvenation, it may be more suitable to aim to maintain general homeostasis and function by appropriately improving immune-inflammatory-functions.

## Introduction

Aging is one of the most intricate and complex biological phenomenon. A comprehensive understanding of aging requires an integrated approach of all physiological systems (1–3). This has captured human imagination from immemorial centuries and the search for a “Fountain of Youth” is still ongoing. Aging is often termed “senescence,” which literally means to grow old. Despite this clear and simple definition, the common interpretation of senescence and related senescent states is shadowed with a negative connotation associated with growing old, that is the only aspect which is inescapably considered is death instead of the process *per se*.

One physiological system that shows marked changes during aging is the immune system (4–7). The interest of the immune system in aging is related to the fact that this is an interacting master regulatory system that keeps the organism free of invaders, either internal or external. Since the introduction of the notion of immunosenescence, many scientists have questioned the justification for unidirectional implication of the immune system and its decreased efficiency associated with aging (8). Whereas some functions are indeed decreased, others are increased. Therefore; changes are not as uniform as the designation would suggest. In this review, we will describe recent advances in the domain of changes in the immune system with aging and outline our vision on how these changes can be dynamically reconsidered from immunosenescence to immunoadaptation/immunoremodeling.

## Immune Changes with Aging: Immunosenescence and Inflamm-Aging, as the two Sides of the Same Coin

The prevailing current opinion is that the most marked changes that occur with aging in the adaptive immune system determine the state of immunosenescence (9–11). It is of note that since the 1980s it has been recognized that the innate system is influenced by aging but perhaps not always in the same direction (12–14). At the turn of the century, a new concept has been put forward by Claudio Franceschi. This concept suggested that aging was associated with a chronic, sterile, low-grade inflammation called inflamm-aging (15). The first related question that arises is what are the characteristic aging-associated changes in the various compartments of the immune system? Furthermore, are these changes faithful indicators of senescence (and progressive incapacitation of the system) or an adaptation, as well as ultimately whether they have any clinical significance. The second question is what the relationship between immunosenescence and inflamm-aging is and how it can be integrated into the broader mechanism of the aging process? Finally, the third question is whether we should attempt to intervene to modulate it.

### Innate Immune Changes

The innate immune response is the most phylogenetically conserved protection in the animal kingdom that allows the organism to efficiently defend against an impressive number of aggressive pathogens (16). This compartment is meant to recognize and react to the conserved pathogen-associated molecular patterns (external threats) and danger-associated molecular patterns (internal threats) by way of specific receptors that play a key role in elimination of the aggressors (17, 18). There are three classes of pattern-recognition receptors (PRRs), each one having distinct roles although all of them elicit some form of inflammation. The first class of PRR comprises the Toll-like receptors which, using various intracellular signaling pathways, results in NF-κB activation and production of various mediators such as cytokines and chemokines (19). The second class comprises the NOD-like receptors, which are able to stimulate the inflammasome complex and that results in the production of IL-1, IL-18, and IL-33 (20). The third class includes the Rig-like receptors that act through the interferon response elements (21). There are also other receptors which are crucial for the functionality of innate immune cells, such as various Fcγ receptors, chemokine receptors such as fMLP receptors, and complement receptors (14). One of the most important observations of these last years, besides the discovery of PRR, is the fact that the innate immune system possesses some sort of memory which has been termed trained innate immune memory (22, 23). As described by Franceschi et al., the innate immune system may be viewed as possessing the “bow tie” architecture where many signals converge to a few sensors but result in many effectors (24). These coordinated events help to elicit a precise and efficient response.

Whereas many immune changes have been described with aging, we will not describe in details all the aging-associated changes for each immune cell type, as this topic has been comprehensively reviewed recently (25, 26). Collectively, the main characteristics of aging with respect to the innate system are immune stimulation at the basal level on the one hand and, immune paralysis when specific functions such as free radical production are needed, on the other hand (8). This dichotomy was initially proposed to be at the basis of the inflamm-aging concept, which stated that the relatively maintained innate response overrode the more altered adaptive immune response, resulting in higher production of pro-inflammatory mediators. Since that original observation, it became obvious that other processes may contribute to inflamm-aging, such as cell senescence, mitochondrial dysfunction, and microbiota changes (dysbiosis) (27, 28). Furthermore, under some circumstances the effects of oxidative stress were included as part of the process (oxy-inflamm-aging), emphasizing the role of the oxidative stress in the complex mechanisms of aging (29). Whatever the nature and the involvement of the components of inflamm-aging are, it is a fact that the phenomenon results in subclinical low-grade inflammation. For instance, life-long antigenic stimulation by pathogens would maintain this quiescent state of innate immune system activation. The innate immune system can also be stimulated by the so-called internal GARBage system (30). Thus, a heightened inflamm-aging state is produced as a consequence of (1) dysfunctional mitochondria, (2) defective autophagy/mitophagy (disposal of dysfunctional organelles), (3) endoplasmic reticulum stress, (4) activation of inflammasome by cell debris and misplaced self molecules, (5) defective ubiquitin/proteasome system (misfolded/oxidized proteins), (6) activation of DNA damage response, (7) senescent T cells and their senescence-associated secretory phenotype (SASP), and (8) age-related changes in the composition of gut microbiota (dysbiosis) (27–29).

The demonstration of trained immune memory may explain, at least partially, some of the immune aspects of aging (22, 23). Following their response, innate immune cells return to a quiescent state due to epigenetic changes and modulation of cell metabolism alternating between (aerobic) oxidative phosphorylation (OX-PHOS) and anaerobic glycolysis (Warburg effect) (31). A subsequent stimulation (e.g., by the same or different type of pathogen) elicits a faster and higher response than the first one due to trained innate memory (32).

The hypothesis of trained innate memory may, at least in part, explain why aging innate immune cells are in a state of sustained activation (14). This concept is relatively novel and was first observed after a specific stimulation, such as the Calmette–Guérin bacillus (BCG). Even after 3 months following challenge, innate cells (monocyte/macrophages) were still able to sustain a certain “memory” of the initial infection and to react in the absence of BCG to any other stimulation (22). This observation has led to the concept that the innate immune system has a certain “memory,” which was not foreseen from the previous paradigm of the immune system function.

Within the context of the aging innate immune system, it can also be suggested that the sustained trained immune memory is (or may be) leading to a sustained state of activation even in the absence of a specific challenge (6). This memory is likely due to a shift in the epigenetic landscape (epigenome) of the innate cells and fueled by an energetic shift of these cells. As yet, there is no formal proof for the contribution of these phenomena to the basic activation of innate immune cells, but this seems strongly probable. If this was the case, the epigenome and pathways involved in energy production, use, and conservation by immune cells could be targets of choice for immune modulation in the elderly. This possibility may explain the suggestions that macrophages are at center stage of inflamm-aging (15). Macrophages are able to modify their phenotype, produce pro- and anti-inflammatory mediators, and orchestrate many vital functions. Moreover, this phenomenon may to some extent resemble hormesis, providing a possibility to better react after each repeated stimulation (33, 34). Finally, it has recently been reported that not only does a high low-grade controlled inflammation was present in aged individuals (centenarians) but also that inflammation showed a better correlation with longevity than any other parameters, according to two longitudinal studies (35, 36).

The epigenetic clock notion in aging whole-organism has been proposed recently (37). ELOVL2 (elongase of omega 3 and 6 fatty acids) was found to be the most powerful single epigenetic biomarker of aging (38). Furthermore, Franceschi’s group has shown that centenarians and their offsprings are epigenetically younger than one could deduct from their chronological age. According to this study (39), semi-supercentenarians are on average 8.7 years younger than expected based on chronological age, and offsprings of aged greater than 105 years are 5.2 years younger than age-matched controls where DNA methylation age and chronological age overlap. These findings reinforce the idea that epigenome control of the innate trained memory and its possible dysregulation with aging lead to DAMAge by inflamm-aging. Can this be reconciled with the heightened inflamm-aging in centenarians? Perhaps trained immune memory is the key regulated by epigenetic changes. The methylation age observed in centenarians suggests that the heightened inflamm-aging is either not connected to it or, paradoxically, methylation age should be younger. These observations may represent a trade-off between a potentially harmful process which, when under tight control, may remain beneficial. It is tempting to suggest that inflamm-aging may be considered the essence of life and the real “Fountain of Youth.” In this context, centenarians may be considered as the standard and not the exception and may serve as model for the better understanding the role of inflammation and epigenetics in aging.

Chronic challenges during aging are paralleled by intracellular changes such as mitochondrial dysfunction, altered autophagy and changes in DNA repair mechanisms. However; immune cells are constantly maintained in an alert state due to chronic low-grade inflammation. However, this state may be counter-balanced by anti-inflammatory molecules as shown in the case of centenarians (40–42). Chronic low-grade inflammation (inflamm-aging) is a physiological response to the life-long antigenic stress and represents an efficient defense mechanism as long as it is under control. Without the essential counter-regulation by anti-inflammatory molecules as seen in aging, it is now clear how damaging this physiological state may be to the whole organism (43). Centenarians seem to represent an exception to the inflamm-aging effects on physiological aging or, perhaps, they present the physiological dynamics of aging. Thus, we may suggest that the norm (successful) aging is exemplified by centenarians whereas individuals that do not reach this age are the biological exceptions that lack the individual epigenetic history and machinery to reach that pinnacle.

The corollary of chronic low-grade inflammation is the downregulation of the innate immune functions or immune paralysis or eventually a sort of innate immune tolerance (8). This physiological condition protects the organism against further self-induced damage even if it is at the expense of the defense from pathogens or from GARBAge. However, it is a reductionist view to assume that this immune paralysis is equal to a non-functional state. Although there are functional alterations in elderly individuals when compared to young subjects, a straightforward assumption that immune cells of elderly humans lose their protective functions. For instance, most elderly humans are able to defend against many types of infections even if the adaptive immune response is somewhat less functional. However, there are relatively few longitudinal studies concerning innate immune function changes with age. Therefore, we do not know whether this is a continuous phenomenon or whether they remain stable during aging. We can speculate that incongruent results of cross-sectional studies suggest that there is not a uniform decrease either related to cell type or to immune cell functions.

Could there be any advantage associated with innate immune paralysis? It can easily be conceptualized that maintenance of identical intensities in the innate cell functions in elderly subjects to the level of young individuals would be energetically very difficult. For instance, this is illustrated by the situation of the large amounts of energy needed for maintaining a M1 (pro-inflammatory and anti-cancer) phenotype in the case of macrophages (44). On the other hand, the M2 phenotype (healing, promoting angiogenesis and cancer growth) consumes much less energy and, therefore, is not as much affected. A decreased production of free radicals can be considered harmful for the eradication of pathogens. However, low free radical production may protect the whole body against further age- and oxidative stress-related damages. Decreased chemotactic activity may also be suggested to be harmful for pathogen destruction, although a sterile inflammatory process may protect against excessive tissue damage. Thus, the question is within the context of evolution, what is most rewarding for the body in an aged organism? On the one hand, is it to destroy pathogens at any cost? On the other hand, is it to maintain physiological integrity by way of chronic inflammation? This dichotomy can mirror recent findings on mitochondria where a certain degree of dysfunction was linked to successful aging and longevity, in contrast to normal or excessively altered functioning in unsuccessful aging (45). This mild impairment may work as a hormetic signal (46). Although there is at present no definitive answer to this question, it illustrates the fact that a broader perspective is needed to understand changes in the innate immune system with aging.

The innate immune system influences the adaptive immune response in many ways. One of these cases is antigen presentation by dendritic cells (DCs). There are conflicting results in this domain and it seems that DCs are less able to prime CD4^+^ T cells in the elderly (47). It is not clear whether the problem is related to antigen presentation or to reaction to antigen presentation. In likelihood; both aspects may be affected by aging. The processing of antigenic peptides by the immune proteasome may not be so efficient and perhaps either the T cell receptor (TCR) itself or TCR-dependent-signaling could be altered (48, 49). The interaction may also occur through an interplay with the cytokines that are secreted by the innate immune system cells. Increased levels of pro-inflammatory cytokine production by the innate cells during aging may also influence the reactivity of the CD4^+^ T cells; e.g., the increased amounts of TNFα may downregulate the expression of CD28 which will negatively affect clonal expansion (50). Moreover, these cytokines may elicit increased free radical production in T cells, which will paralyze their function by increasing inhibitory events of signaling (51). In sum, alterations in the innate immune system may also impact adaptive immune changes with aging.

### Adaptive Immune System

The adaptive immune system is composed of the cellular and the humoral immune response. T cells are orchestrating the cellular immune responses. These cells are basically divided into CD4^+^ and CD8^+^ T cell populations, which possess very clearly defined functions. CD4^+^ T cells are helper cells that regulate the functions of all the other immune cells. They also possess effector functions (52). CD8^+^ T cells are effector and memory T cells responsible for clearing the aggressors (53). The CD4^+^ compartment may be subdivided, taking into account functionalities, in Th1, Th2, Th17, and regulatory T cell (Treg) subpopulations (54). Phenotypically, CD4^+^ and CD8^+^ T cell compartments are subdivided into four functionally distinct subpopulations, which are naïve, central memory, effector memory, and T effector memory cells re-expressing CD45RA (TEMRA) (55).

Many alterations in the adaptive immune system have been described in aging (11, 56, 57). With respect to T cell subpopulations, aging is characterized by two main changes: a decrease in naïve T cells that leads to the shrinking of the TCR repertoire and an increase in memory T cells that is primed by different aggressors. Recent thymic emigrants of new naïve cells are vanishingly rare in the elderly because of thymic involution at puberty and acute and chronic antigenic stress over the lifetime and, age-associated hematopoietic stem cell insufficiency (9, 10), This phenomenon is considered as one of the most basic changes in the adaptive immune system with aging. How does this situation happen? This seems to be the main explanation for the increased incidence of infections, cancers and the failure of vaccination in elderly (58–60). These observations mean that elderly individuals are less able to respond to neo-antigens than young individuals. However, this idea has been seriously challenged in recent years, mainly on the basis that there may not be a dramatic shrinkage of the TCR repertoire involving the remaining and slowly produced new emigrants, as supposed for decades (60). Moreover, the newly reconsidered homeostatic proliferation of naïve T cells under IL-7 stimulation may replace the failing thymus, at least partially. The recently discovered Stem Cell-like Memory T cells may also participate in incomplete replenishment of the naïve T cell compartment (61). Overall, the alterations may not be so dramatic and even the T cell repertoire may be relatively sufficient to supply the demand. Indeed, centenarians do not present more cancer, as its prevalence plateaus after the age of 90. Furthermore, there is no tendency for these individuals to suffer from unknown new pathogen-induced infections. Findings of two longitudinal studies led to the conclusion that having more CD8^+^ naïve T cells was not considered a survival advantage (62, 63). These new data shed serious doubts on the present concept of “immunosenescence,” at least in the adaptive compartment. However, the debate between immunologists and gerontologists is far from being settled.

There could be some evolutionary reasons for thymic involution. First, the maintenance of an organ so metabolically active may be very resource-demanding in the situation where the whole organism tends to reduce energy consumption during aging. This phenomenon can parallel the other two very energy-demanding organ shrinkage situations seen in aging, namely those of muscles and bone marrow. Second, during life, the organism has already encountered most of the pathogens typically active in the temporal and spatial region of its dwelling. Thus, resources must be allocated preferentially to combat these “usual,” cognate pathogens by the memory part of the immune system rather than spend energy for a useless fight, which may be terminated in any event by destruction of the invading organism.

Thymic involution is a double-edged sword. On the one hand, it may indirectly be responsible for the death of the organism which would then lack the right TCR to mount an effective response against neo-antigen(s). On the other hand, it results in lower energy consumption which becomes available for other survival-supportive functions and activities of the organism. Given the relative rarity of direct infectious causes of death in the elderly, it would appear that downregulation of capacity to respond to novel pathogens during aging does not come at an excessive cost.

The increase in the number of memory T cells may be very rewarding for the aging organism as this will continuously assure survival against attacks by cognate pathogens that may threaten the survival of the organism. T cells are all directed against specific internal and external aggressors. The body hosts many latent infections which can re-activate from time to time under specific conditions (55). One well characterized pathogen of this type is the cytomegalovirus (CMV) (62). CMV was once considered the main cause of age-related immune changes in the elderly. Although accumulating data are still quite contradictory, the current belief is that the presence of CMV infection does not seem to be only detrimental (63–66). On the contrary, CMV infection may be considered a recurrent stimulation that maintains a sustained immunological alertness that favors a better immune response, e.g., to vaccination (67). The global response to the many various CMV antigens has even been linked to better survival (68). Thus, the increased number of committed memory T cells may not be considered unequivocally as detrimental or related only to aging.

One of the most important features of aging is the notion of senescent cells (69). This idea has re-gained popularity in recent years as a way to explain the decreased functionality of the immune system with aging (70). Senescent cells conform to the model of Hayflick replicative senescence as they are not proliferating but remain metabolically active and secrete several pro-inflammatory substances (SASP) (71, 72). Formerly, accumulated memory T cells were considered “senescent” (70). However, experimental evidence suggests that these cells are still able to function when pathogens such as CMV are re-activated (64, 65). Furthermore, there are no universally accepted markers of cell senescence (7). Finally, cell senescence is also a double-edged sword as these cells are needed in the case of some physiological functions, for instance repair and fight against cancerous transformation, whereas they are detrimental—to other cell functions (73, 74).

There is also a large confusion in the field of aging with respect to the number and distinction between senescent and exhausted cells (75–77). Senescent and exhausted immune cells are to be distinguished as the former may be functionally inert, whereas the latter may be functionally “dormant.” This distinction is crucial when considering immune functions in relationship to aging. Exhausted T cells can be awakened by modulation of some surface receptors called the immune checkpoint inhibitors and, they can then resume function (78, 79). The most important of these receptors are PD-1, CTLA-4, LAG-3, TIM-3, and TIMIN. This distinction has gained considerable importance since it has been reported that a number of cancers in some elderly subjects could be successfully modulated and T cells engineered to be immunotoxic toward such cancers, namely melanoma and NSCLC (80–82).

One additional aspect where the literature has not, in our view, paid enough attention is the age-associated impairment of metabolic regulation of immune cell functions which is of vital importance for an adequate immune response. Quiescent cells compared to activated cells require different metabolic responses (83–87). Whereas quiescent cells use the OX-PHOS pathway for their functions that generates 36 ATP per metabolized glucose, activated cells use anaerobic glycolysis, which generate two ATP. Why is it so? When cells are activated, they need energy very quickly which cannot be provided by the OX-PHOS pathway, but only by aerobic glycolysis (Warburg effect). This is another example where the body is trading efficiency for rapidity, as in many circumstances in the aging immune system. Not only do the quiescent and activated states have different metabolic requirements but also the differentiation of the various subtypes of T cells is dependent on the specific metabolic pathways used (88). The master of cellular metabolism is the mTOR pathway that regulates clonal expansion, whereas its inhibition drives (*via* autophagy) the reconstruction of damaged cells (89). Very few studies in aging have addressed the metabolic changes that occur in immune cells with aging (90). However, one of these studies has emphasized the metabolic differences in T cells of young and elderly subjects (91). T cells of elderly individuals suffer from insufficient substrate to feed mitochondrial respiration and, consequently, are energy deprived. Instead of breaking down glucose, they shunt it into the pentose phosphate pathway, promoting an anabolic state. One of the metabolic consequences is the accumulation of reductive elements, particularly NADPH and reduced glutathione, and the scavenging of reactive oxygen species (91). Energy-deprived T cells upregulate activation of the energy sensor 5′-AMP-activated protein kinase (AMPK). A downstream target of inappropriately activated AMPK in aging T cells is the dual-specificity protein phosphatase 4 (DUSP4) (92), which negatively regulates members of the MAPK superfamily, in particular ERK1, ERK2, and JNK. ERK is also subject to increased negative regulation by another dual-specificity protein phosphatase, DUSP6 (93). ERK is a key regulator of the T-cell receptor signaling cascade, and its dephosphorylation by DUSP4 and DUSP6 functions as a suppressive mechanism, weakening the TCR-induced signal and dampening T-cell function. Thus, it may be very important to take into account age-related changes to determine how the extrinsic nutritional availability of glucose, amino-acids, and lipids will modulate age-related changes in immune system functioning.

Once immune cells are stimulated as a result of recognition of cognate antigen presentation, they initiate signaling pathways that result in the transcription of the appropriate molecules required for the expected functions (94–96). In elderly subjects, this cascade of events has been found to be altered, from impaired immune synapse formation to defects associated with translocation of transcription factors (49). Interestingly, detailed investigations of these alterations have revealed that they were mostly related to factors which could be considered modulable. One of the key factors that are involved in these changes are the composition and the organization of components of the plasma membrane, which orchestrates assembly of signaling molecules in cholesterol/ganglioside-containing nanoclusters (97). Thus, changes that were once considered a part of the aging process could be viewed as only the manifestation of some environmental interference and modulated by lifestyle factors such as exercise and nutrition (98).

## What is the Relationship Between Immunosenescence and Inflamm-Aging?

According to the original concept of inflamm-aging, a consequence of immunosenescence, the relatively conserved innate immune system overtakes the more altered adaptive immune system in aging. However, recent data are more in line with the interpretation that this is not a unidirectional relationship, but a mutually maintained state where immunosenescence is induced by inflamm-aging and *vice versa*. The main changes in the aging adaptive immune system occur in the T cell compartment (57, 91). There is an increase in the number of memory CD8^+^ T cells, which were originally considered relatively non-functional (99). These cells are characterized by the loss of naïve T cell surface markers, such as CD28, CD27, and the emergence of new senescent markers such as KLRG1. It has been found that the increase in the number of memory T cells and, later on, that of B cells may be due to a continuous chronic antigenic stimulation similar to the phenomenon of inflamm-aging. Infection by CMV emerged above all the large variety of potential stimulating agents (62). However, some data indicate that CMV infection could not be differentiated from inflamm-aging between seropositive and seronegative individuals (100). It is conceivable that the body devotes a huge part of its immune resources to contain this specific infection throughout life. Consequently, the immune space becomes filled with CMV-specific memory CD8^+^ T cells. These cells have been previously considered to be inactive but recent data have shown that they are metabolically active and their senescent phenotype (SASP) can participate in the development of inflamm-aging (76). Thus, chronic antigenic stimulation leads both to the phenomenon of inflamm-aging and the increase of the number of senescent T cells. One additional consequence of chronic stimulation is the phenomenon of exhaustion, characterized by the emergence of inhibitory receptors, such as PD-1, CTLA-4, and many others (75). Other cell types of the adaptive immune system are also affected by aging but to various extents. For instance, the CD4^+^ T cell population also undergoes similar changes to CD8^+^ T cells but to a different extent (55). The Treg population also increases with aging as well as the pro-inflammatory Th17 subpopulation (101). Finally, the B cell compartment is also altered with aging (102). The functional consequences of these overall changes result collectively in the decreased ability to fight new challenges. Thus, clonal expansion, cytokine production, and specific antibody production are compromised. This situation leads to increased infections, cancer, and chronic diseases in the elderly (43). It appears that inflamm-aging and immunosenescence progress in parallel and form a vicious cycle. Increased production of inflammatory mediators characteristic of inflamm-aging contributes to the decrease of the adaptive immune response and, eventually, to immunosenescence. In contrast, the decrease of the adaptive immune response reinforces the stimulation of the innate immune response (as the means to protect organism from infections in the circumstances when adaptive immunity fails) leading to inflamm-aging. Both processes are important not only as causes of immune changes in the elderly but also (or even mainly) because of their consequences in the aging organism.

## Immunosenescence/Inflamm-Aging; Why Does it Matter?

One can ask why all these changes in the immune system with aging do matter. The paradigm for many years has been that immunosenescence and inflamm-aging are the fertile soil for the development of diseases mostly considered as age-related, either acute such as infections, or chronic such as cancer, frailty, Alzheimer’s disease (AD), and cardiovascular diseases (CVD) (43). The bulk of these observations has led to the field of geroscience (103–105). The consequence of this new field leads to a novel approach that consists in targeting the aging process as the single most important risk factor instead of treating each disease separately. This notion should be nuanced by individual aging, suggesting that all individuals do not age in the same way and perhaps the underlying mechanisms may be different (6).

Alterations in T cell functions, more precisely the decrease in the number of naïve T cells and the increase in number of memory T cells, has been considered the main explanation for increased incidence of infections and cancers in the elderly (43). However, there is still no direct evidence from experimental observations or longitudinal studies, which could really support this hypothesis. It is of note to mention that the overall incidence of malignancies decreases after the age of 90 (106). However, would the incidence of infections, which is claimed to increase; still be true if one would only take into account elderly individuals with healthy aging? It is also of note that relatively few elderly subjects die of infections, even if severe and requiring hospital treatment. For example, in Canada in 2013, only 4.1% of deaths in individuals aged more than 65 years could be directly attributable to infectious causes (International Classification of Diseases A00-A99, B00-B99, G00-G03, J09-J21) (Statistics Canada: http://www5.statcan.gc.ca/cansim/pick-choisir?lang=eng&searchTypeByValue=1&id=1020561, accessed Oct 23, 2017).

It has also been commonly believed for decades that elderly individuals responded poorly to vaccination, which sometimes led to generalized doubts about the efficacy of vaccination in old age in general. Among the many vaccines which were considered less efficient (107), influenza vaccination was generally cited as the gold standard (108, 109). It is now established that there are many factors besides immunosenescence, which influence effectiveness of this vaccine and others. In fact, knowledge and better understanding of these factors has already led to enormous enhancement of efficacy of vaccination (e.g., against herpesviruses) (110).

Inflamm-aging could play a role in the late manifestation of diseases such as AD, frailty syndrome (FS), and CVD. It is of note that, besides FS, the onset of these diseases start in young or middle age when the immune system is still efficient, that is before signs of immunosenescence or inflamm-aging are detectable. Frailty can be considered a manifestation of (unsuccessful) aging and may represent the clinical sign of biological age, in contrast to chronological age (111). Furthermore, in a longitudinal study, inflammation was found to be the most important factor to account for longevity, especially in semi-super centenarians (36). In conclusion, there is no doubt that immunosenescence and inflamm-aging contribute to the increased incidence of age-related diseases. However, their exact role is not yet well defined, and in some case, it may be even doubtful. There has been little exploration of the possibility that there are optimum levels of immunosenescence and inflamm-aging and that too much or too little could exacerbate the risks of various diseases in the elderly.

## How can Successes in Vaccination and Immunotherapy be Interpreted in Light of Immunosenescence?

There has been recent reports of therapeutic successes in domains which were strongly considered to have the potential to overcome immunosenescence, namely the decreased immune response of the elderly to vaccination and the failures in treatment of some cancers. For example, in one study a new vaccine was tested for herpes zoster (shingles) prevention. This study showed that the administration of the antigen in combination with an adjuvant induced a strong immune response even in subjects older than 80 years of age (110). Furthermore, protection by this vaccine was high even after the 3.2 years of follow-up. This report should serve as a lead to question whether reported lack of vaccine efficacy in the elderly is due to immunosenescence or to improperly designed vaccines. Alternatively, even if a decrease in the immune response occurs it may be overcome by a well targeted vaccine (110). Recently, there has been a rise of immune checkpoint inhibitors as effective therapeutics in cancer treatment. While still sparse, the observations of effectiveness in elderly subjects are very encouraging. For example, in the case of metastatic melanoma, the use of Nivolumab^®^ and Ipilimumab^®^ either alone or in combination had survival effects in elderly subjects similar to young patients (80–82). This is a remarkable result that suggests that the exhausted T cells of the elderly are still able to respond to inhibition of their inhibitory receptors with a recovery of cytotoxic activity. It is to be mentioned that immunotherapy often works well in the elderly, but the treatment has to be adapted to the patients and be different than for young people (112). What works for one does not necessarily work for the other, but this does not mean that the elderly will not respond. The longevity advantage in some cases of CMV infection may also militate against the exclusively detrimental effect of the immunosenescence and inflamm-aging (68).

## How Should we Interpret Immunosenescence and Inflamm-Aging?

We suggest that there is a need for a complete reconsideration of immune changes with aging to gain access to a better understanding of their mechanisms and to ensure that eventual interventions do more good than harm. The question does follow: which immune changes in the elderly may be beneficial? The answer to this question would require careful reinterpretation of current data, but it could also be highly useful to cope with an extended perspective of aging. There are several potential changes in the adaptive immune system with aging. First, increased proportions of adaptive memory cells may be beneficial to fight cognate pathogens more efficiently. Thymic involution may be considered as needed for reduction of energy consumption by an organ which is not absolutely necessary for survival and the maintenance of which is energy-costly. Finally, the increased proportions of Tregs observed in the elderly may prevent autoimmune onslaught.

There are some alterations that may not be solely detrimental in the innate immune system. For instance, increased proportions of innate (“trained”) memory cells may help to efficiently fight cognate—and some not so cognate—pathogens. Increased asymptomatic pro-inflammatory state (conceptualized as increased “readiness” of innate immunity to pathogen challenge) may have some evolutionary advantages and could even be considered necessary if it is well regulated, i.e., not excessive. However, too much of a good thing could ultimately lead to disastrous consequences at any age, e.g., free radicals.

**Figure 1:**
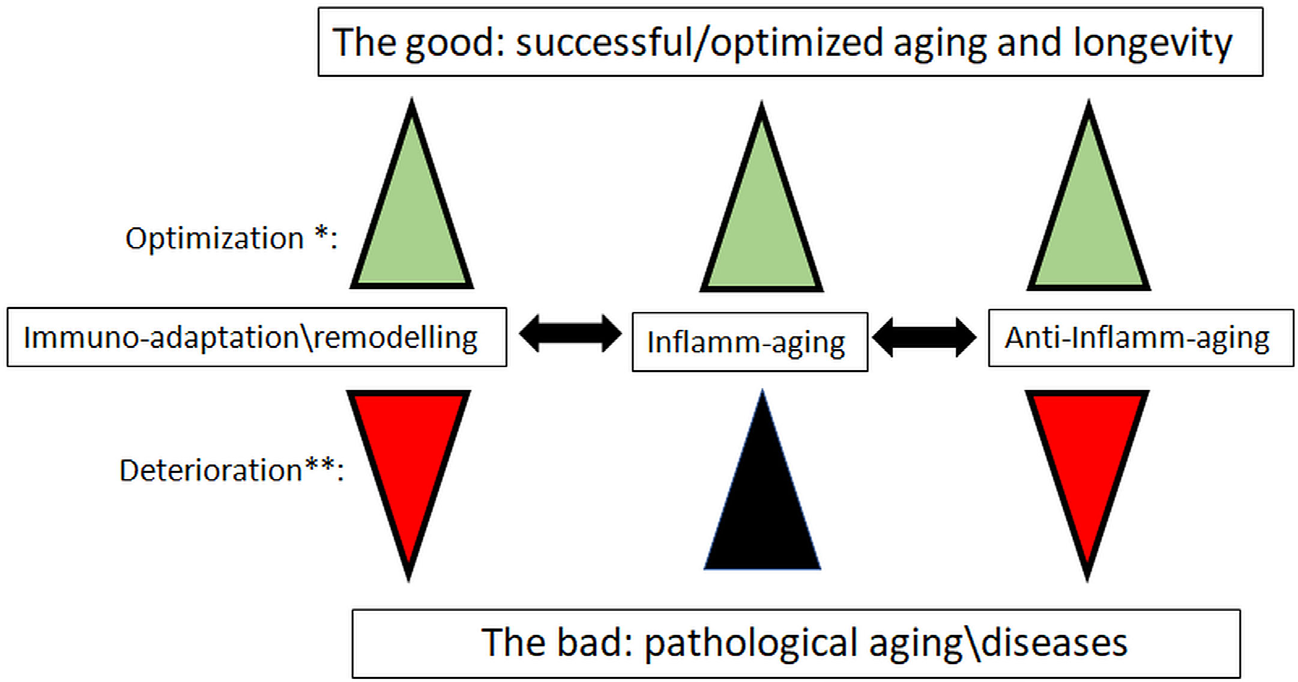
The new paradigm for the role of inflamm-aging and immunoadaptation/remodeling in the aging process. *Optimization: all three processes increase in concert, balancing each other. **Deterioration: inflamm-aging increases, and is not balanced by opposite processes of anti-inflamm-aging and immune-adaptation/remodeling, which are decreasing. We mean by anti-inflamm-aging all compensatory mechanisms which emerged to compensate the chronic inflamm-aging. The most important diseases that could have an inflamm-aging component are cancers, cardiovascular diseases, and neurodegenerative diseases.

Accordingly, we can propose a new paradigm for dynamic immune changes with aging (Figure 1). We suggest that aging leads to modified/modulated responses of the immune system, making it more adapted to cope with challenges (pathogens) in a given (local) environment, and not just to an eventually terminal deterioration of the immune system. From an evolutionary perspective, this is a simple optimization of the resources of the aging body, even if it ultimately leads to pathologies and death. From this perspective, many or most age-related changes in the immune system may be desirable adaptations to the aging process, and thus no need for rejuvenation seems to be necessary.

## Two Important Recent Approaches to Better Understand Immune Changes with Aging, Supporting the Need for a Change of the Current Paradigm

There are two new approaches which can be adopted to re-conceptualize immune changes with aging. These integrate almost all aspects mentioned above. Immune systems of the elderly are remodeled with fewer naive cells and dysfunctional (exhausted vs. senescent) memory cells, due to chronic antigenic stimulation (including, but not limited to, CMV and neo-antigens from emerging malignant cells) and thymic involution, with altered innate immune response resulting in inflamm-aging eventually contributing to some age-related disease development (Table 1).

**Table 1:** Summary of some immune changes associated with aging in innate and adaptive immune systems.

Features	Increase	Decrease	No change
**Innate immunity**			
Phagocytosis	−	√	√
Free radical production	√	√	−
Chemotaxis	−	√	−
Cytokine production	√	−	−
Myeloid cell number	√	−	−

**Adaptive immunity**			
Naïve cell number	−	√	−
Memory cell number	√	−	−
T regulatory cell number	√	−	−
T regulatory cell function	−	√	−
Proliferation	−	√	−
IL-2 production	−	√	−
B regulatory cell number/function	−	√	−
B cell immunoglobulin production	−	√	−
B cell autoantibody production	√	−	−

*Changes are indicated with a checkmark (√) and, absence of changes with a horizontal bar (─)*.

### How do Inflamm-Aging and Immunosenescence Stand from an Evolutionary Perspective?

Considering all the alterations in the immune system with aging, the question arises whether and how inflamm-aging and immunosenescence can be the cause of these numerous age-related alterations and pathologies attributed to them. Recent observations tend to challenge the established view of the role of immune changes with aging. The publication of Lal et al. cited above that reported a positive response to a new herpes zoster vaccine even in the very old raises the question of the role of immunosenescence and inflamm-aging in the decreased response to vaccination (110). An additional recent report has further suggested that inflammation is a driving force for longevity in super semi-centenarians (36). In another study (113), Franceschi’s group determined HCMV prevalence in 132 centenarians, 245 centenarian offspring, and 101 offspring of non-long-lived parents. These authors found that infection did not impact on the longevity of these elderly individuals. Finally, the studies concerning the diversity of the microbiota in centenarians may also support this changing paradigm as dysbiosis is not always the equivalent of dysregulated inflamm-aging, particularly in centenarians and semi-super centenarians. The changes in the composition of the gut microbiota with age in subjects ranging from 22 to 109 years can be mentioned as one of the best example of remodeling, with possible large influence on inflamm-aging and immunosenescence, taking into account how important is the gut microbiota for the immune system. An increase in sub-dominant species and among them, species which are considered very “good,” was observed in Italian, Japanese, and Chinese centenarians despite the differences of diet and genetics in aged subjects, particularly in semi-supercentenarians (114, 115).

This situation did not involve a chronic uncontrolled inflammation, but the well-balanced inflammatory and anti-inflammatory equilibrium. Immune changes observed during aging may thus only represent an adaptation to a challenging environment (containing mostly the cognate pathogens, with the exception of cancer cells generated by semi-random mutations) that results in maintenance of homeostasis *via* hormesis. However, under conditions of not previously encountered pressure (i.e., contact with a novel, previously unknown pathogen), the aging immune system either can adapt by using the available reserves. Conversely, if it is unable to do so, that leads to a maladaptation manifested by age-related diseases or a lethal outcome.

From this perspective, it is interesting to consider the putative role of inflamm-aging in frailty. The definition of frailty is already controversial as two main designations exist, namely the phenotypic definition (116) and the multiple composite burden (deficit accumulation) (117). Frailty in fact can be conceptualized from an evolutionary point of view as the decrease of the physiological/biological/molecular reserves of the aging organs/organism, leading to less efficient responses to stresses and, therefore, producing deleterious effects, even death (4, 111). This could be considered normal (usual) aging, in contrast to successful aging on the one hand or pathological aging on the other hand. Independently of its definition, one of the most accepted causes of frailty is inflamm-aging (4, 118) that represents the biological threshold between successful and pathological aging. This event suggests a dynamic process that could still be reversed if the underlying causes such as inflamm-aging were contained. However, this situation may also progress to death through diseases when it becomes uncontrolled and hyperinflammatory, as would be predicted by the trained innate memory process (8). From this perspective, the extent of symptoms included in the clinical picture of frailty may be considered as a surrogate measure for biological age independently of chronological age, indicating whether inflamm-aging tends toward health or disease/vulnerability. Then an “optimal inflamm-aging” may be defined for longevity and health (optimal aging). Eventually, as is the case with all biological processes, an equilibrium is needed with functional checkpoint gatekeepers. If, for any reason, this equilibrium is perturbed the pathological pathway may prevail. The aim of optimization efforts and approaches would be to help maintain this very complex equilibrium to achieve an adequate functional longevity for optimal aging.

### A Dysregulatory Approach Integrating the Immune System and Other Systems

Clearly, the immune system does not exist in isolation but is influenced by and, in turn, influences many other systems such as the central and the peripheral nervous system, the endocrine system and others (3, 4, 119). This fact fits perfectly with the new approach of the study of aging which states that aging is the sum of results of the dysregulation of different system(s) from a normal regulatory level (i.e., a homeostatic state). This state is not necessarily identical between young and old subjects and may well reflect adaptations to intrinsic (GARBAge) and extrinsic changes (mainly pathogens and adverse environmental influences) related to aging. Thus, dysregulations are neither obligatorily detrimental nor beneficial but indicate a state of dyshomeostasis. This statement leads to the important insight that the pro-inflammatory state cannot be considered separately from the anti-inflammatory state (41). In this respect, this possibility will likely be expanded and nuanced by inclusion of other systems and cellular subsystems, e.g., mitochondrial metabolism. More broadly, this approach suggests that there are clear limits to the relatively linear, pathway-based, reductionist approaches to understanding physiology in general and immunology in particular. “Optimal” levels of various cell types, surface markers, and cytokines are unlikely to be very high or very low, but often intermediate, suggesting non-linear associations with risk. These optimal levels are also likely to vary depending on many other factors, such that it will be quite tricky to identify generally healthy or unhealthy states. Because of the possibility that changes with age or health state represent adaptations rather than aspects of pathology, substantial care must be exercised when interpreting changes in the system.

This way of thinking is a completely innovative approach which can be studied by various statistical analyses using smaller or larger databases obtained in the studies of elderly subjects. This approach can also lead to the discovery of new biomarkers and their use in clinical settings. Such a broad approach would allow the integration of the omics, single cell assessment and systems biology. Different statistical approaches may be warranted both in cases when the dysregulation(s) follow a single unidirectional pathway (as appears to be the case for inflamm-aging and likely metabolic syndrome) (119, 120), as well as in cases where dysregulation can produce a wide array of phenotypes sharing little beyond their departure from a homeostatic state (121–123). An appealing hypothesis is that canalized dysregulations occur as a result of adaptations to the aging process and thus reflect an optimized response to an imperfect situation. In contrast, non-canalized dysregulations reflect a true loss of homeostatic control and may themselves be the imperfect situation causing the canalized responses.

Identification of cell types in both the innate and adaptive immune systems that are affected by age-related changes would be an additional application of these kinds of integrative statistical approaches. Immunology has generally considered individual cells to belong to discrete types that can be distinguished based on their surface markers. Certainly, this is a valid paradigm for many of the major classes of immune cells (T-cells vs., B-cells, CD4^+^ vs. CD8^+^, etc.), but many examples of less distinct, partially overlapping classes are starting to emerge: classes based on the levels of surface receptors rather than just their presence or absence, or classes confounded by some subpopulations that are simultaneously expressing two markers that were supposed to distinguish populations (e.g., CD45RA^+^ vs. CD45RO^+^). It would thus appear that variation in cell surface receptors can happen in different ways. Discrete variation produces populations of cells with distinct functions and properties (“classes” or “types”), whereas continuous variation produces cells with functions and properties that vary along a gradient. This distinction is important because if one wrongly considers cells varying along a gradient as being from discrete classes, one is likely to (a) misidentify many cells with intermediate phenotypes and (b) to fail to understand the true biological processes driving cell diversity, thus wrongly interpreting the functional consequences of this diversity. For example, one can suppose that a population of cells varies along a gradient that determines their affinity for two types of pathogens (say, A and B). The cells with high affinity for A would have low affinity for B and *vice versa*. If there were a true gradient, a reasonable strategy would be to have a large population of cells with an intermediate affinity for both, maximizing the flexibility of the response. This may be a particularly good strategy during aging when the total cell population declines and the ability to maintain large numbers of cells at both extremes of the gradient is compromised. If one wrongly divides cells into A-affinitive and B-affinitive types, one may obtain uninterpretable or confusing results, depending on where along the gradient the threshold is set. It would then be not possible to understand any strategies that involve the use of intermediate values along the gradient.

### Should We Intervene and How?

If we consider immune changes related to aging as an adaptation/remodeling, interventions are presently very difficult to foresee. In particular, a single, generalized immune intervention does not appear to be likely. Anti-inflammatory interventions may depend on the state (level) of inflammation and its duration and, on interactions of the innate immune system with other systems, as well as on the appropriate inflammatory state of the individual given age and disease status. If one assumes that the immune/inflammatory system in the elderly/aged organism is adapted/remodeled in order to provide the best possible anti-pathogen protection when the adaptive immune system fails, the rejuvenation approach as currently proposed (e.g., IL-7/IL-15) seems likely to cause potential long-term harm in the aged organism.

Perhaps, more general, however, purposeful interventions may be necessary, such as lifestyle interventions with personalized exercise and nutrition. Specific epigenetic diets may have their role in this modulation (124, 125). Some drugs with global action have been suggested to decrease the (over)activation of the immune/inflammatory system. One such drug is metformin, suspected for a long time to be a powerful modulator of aging (126). Still, its effects in aging immune/inflammatory system are not yet clear. In any case, any interventions will need to be personalized and the immune history (immunobiography) of the individual will have to be taken into account.

## Conclusion and Future Perspectives

Aging is a highly complex process but an increased understanding of the process should lead to the efficient treatment of the many age-related diseases. The immune system interacts with many other systems in the organism (mainly the neural, metabolic, and the endocrine systems) and is, therefore, one of the most ubiquitous master systems of the organism. As such, it orchestrates health when it functions well but, when maladapted, it leads to diseases in the aging organism. Many changes in the immune system with age have been described and most of them have been considered deleterious and causes of many age-related diseases. Changes occur in both the innate and the adaptive immune arms of the immune system, but perhaps not to the same extent or with the same consequences. There is an intricate interrelationship between inflamm-aging and immunosenescence, which are nearly identical in some ways but very different in other aspects and, occurring in concert, mutually influencing each other. Future studies are obviously necessary to elucidate these interactions and raise targets for new interventions to decrease the deleterious effects of aging and use the beneficial effects for a better health and functionspan in the elderly.

Therefore, the phenomenon traditionally termed “immunosenescence” may be considered an immunoremodeling/adaptation as a result of chronic aggressions and time. Immunosenescence may be necessary for an adequate response to known antigens, but detrimental for responses to new antigens in most circumstances. The discovery of new processes, new immune cell subtypes, and the integration of genetic/epigenetic/metabolic and environmental factors (nutrition) will nuance our «evil» and apparently mistaken perception that aging-associated immune changes are only detrimental. Immunosenescence/inflamm-aging may contribute to diseases such as cancer but its role during aging is still controversial. Elderly in clinical settings are doing much better than predicted from experiments thus, human studies in particular are badly needed.

In view of the successes of cancer immunotherapy and vaccination in the elderly, no such intervention should be refused to an elderly subject based on a dogmatic assumption that aging-related immune changes are detrimental. Thus, time is of the essence; and the future is already now for the elderly.

## Author Contributions

TF has written and conceptualized the article; AL, GD, AP, EF, AC, JW, and CF have contributed to the writing and critically read it.

## Conflict of Interest Statement

The authors declare that the research was conducted in the absence of any commercial or financial relationships that could be construed as a potential conflict of interest.
